# A Motif in the F Homomorph of Rabbit Haemorrhagic Disease Virus Polymerase Is Important for the Subcellular Localisation of the Protein and Its Ability to Induce Redistribution of Golgi Membranes

**DOI:** 10.3390/v9080202

**Published:** 2017-08-01

**Authors:** Nadya Urakova, Andrew C. Warden, Peter A. White, Tanja Strive, Michael Frese

**Affiliations:** 1 Health and Biosecurity, Commonwealth Scientific and Industrial Research Organisation, Canberra, ACT 2601, Australia; urakova@uab.edu (N.U.); andrew.warden@csiro.au (A.C.W.); tanja.strive@csiro.au (T.S.); 2 Invasive Animals Cooperative Research Centre, University of Canberra, Canberra, ACT 2601, Australia; 3 Health Research Institute, University of Canberra, Canberra, ACT 2601, Australia; 4 School of Biotechnology and Biomolecular Sciences, Faculty of Science, University of New South Wales, Sydney, NSW 2052, Australia; p.white@unsw.edu.au; 5 Institute for Applied Ecology, University of Canberra, Canberra, ACT 2601, Australia

**Keywords:** *Rabbit haemorrhagic disease virus*, RHDV, caliciviruses, RNA-dependent RNA polymerase, Golgi membranes

## Abstract

*Rabbit haemorrhagic disease virus* (RHDV) is a calicivirus that infects and frequently kills rabbits. Previously, we showed that the RHDV RNA-dependent RNA polymerase (RdRp) is associated with distinct, but yet uncharacterised subcellular structures and is capable of inducing a redistribution of Golgi membranes. In this study, we identified a partially hidden hydrophobic motif that determines the subcellular localisation of recombinant RHDV RdRp in transfected cells. This novel motif, _189_LLWGCDVGVAVCAAAVFHNICY_210_, is located within the F homomorph, between the conserved F3 and A motifs of the core RdRp domain. Amino acid substitutions that decrease the hydrophobicity of this motif reduced the ability of the protein to accumulate in multiple subcellular foci and to induce a rearrangement of the Golgi network. Furthermore, preliminary molecular dynamics simulations suggest that the RHDV RdRp could align with the negatively charged surfaces of biological membranes and undergo a conformational change involving the F homomorph. These changes would expose the newly identified hydrophobic motif so it could immerse itself into the outer leaflet of intracellular membranes.

## 1. Introduction

The *Rabbit haemorrhagic disease virus* (RHDV) is a non-enveloped, single-stranded, positive sense RNA virus representing the genus *Lagovirus*, family *Caliciviridae* [1,2,3]. RHDV-infected cells produce two RNAs, a 7.4-kb genomic RNA and a 2.1-kb subgenomic RNA. Both RNAs are polyadenylated at the 3′ end and covalently linked at the 5′ end with the viral genome binding protein (VPg) [4,5]. The genome encodes two structural proteins (VP60 and VP10) and seven non-structural proteins (p16, p23, the helicase, p29, VPg, the protease and the RNA-dependent RNA polymerase (RdRp)) [6,7]. Virus–host cell interactions are poorly understood, mainly due to the lack of a robust cell culture system.

Similar to other positive-sense RNA viruses, the RHDV RdRp plays a central role in the viral replication cycle. RdRps have many enzymatic properties; they bind template RNAs, initiate replication, catalyse elongation and terminate replication [8]. An analysis of calicivirus RdRp crystal structures revealed that the RdRp of RHDV and related viruses resembles the shape of a right hand with fingers, palm and thumb subdomains [9,10,11,12,13].

**Figure 1:**
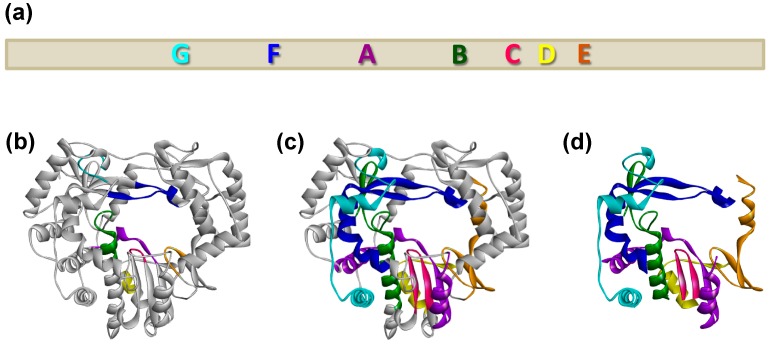
Conserved motifs in RNA-dependent RNA polymerases (RdRps) of single-stranded, positive sense RNA viruses. Conserved sequence motifs A–G are coloured purple, green, pink, yellow, brown, dark blue, cyan, respectively, and their relative positions are shown in a schematic representation (**a**) and a ribbon diagram of the *Rabbit haemorrhagic disease virus* (RHDV) coding sequence (**b**). Conserved structural elements (homomorphs) that encompass the conserved sequence motifs A–G are shown in the context of the entire protein (**c**) and without other protein elements (**d**).

RdRp protein sequences are usually highly variable; strong sequence conservation is observed only for about 20 amino acids that form seven functional sequence motifs, named motif A, B, C, D, E, F and G [14,15,16,17]. Conserved structural elements (homomorphs) that encompass these conserved sequence motifs contain approximately 75% of all amino acids in the protein (Figure 1). Despite considerable sequence diversity, each homomorph has a high structural similarity to related segments in other RdRps [17]. Of the seven sequence motifs, the A, B, C, D and E motifs are located in the palm subdomain and are involved in template binding, substrate discrimination and catalysis [8,18]. Motifs A and C contain catalytic aspartic acid residues that are strictly conserved [8]. F and G motifs are located in the fingers subdomain [15,16,17,19,20]. The G motif is located near the outer edge of the template channel and may enforce the correct orientation of essential amino acid residues and a primer that is required for the initiation of RNA replication [10,17,20]. The F motif has a number of basic amino acid residues that are involved in interactions with incoming nucleotide triphosphates [15,16,17,18]. The presence of an additional conserved structural motif (H motif) in the thumb subdomain was recently proposed [21]. However, none of the amino acids in the H motif are strictly conserved and their exact function remains elusive [15,21].

Recombinant RHDV RdRp, in contrast to related RdRps of other caliciviruses, such as the *Murine norovirus* (MNV), has an unusual subcellular localisation. Hyde and Mackenzie [22] showed that immunostaining of recombinant MNV RdRp produces a diffuse cytoplasmic staining and a somewhat weaker nuclear signal, a localisation pattern that is similar to that observed with RdRps from other viruses, e.g., *Mouse hepatitis virus* [23], *Hepatitis C virus* [24], *Kunjin virus* [25] or *Polio virus* [26]. Surprisingly, our previous studies revealed that only a small percentage of cells expressing recombinant RHDV RdRp proteins showed the usual staining pattern for viral RdRps; in the majority of cells, the RHDV RdRp accumulated in distinct, but yet undefined subcellular structures [27,28].

In this study, we identified a putative functional motif that influences the subcellular localisation of the RHDV RdRp and its ability to interfere with the organisation of the Golgi apparatus. Molecular dynamics simulations suggest that the newly discovered hydrophobic motif is normally buried but can be exposed, enabling direct RdRp–membrane interactions. Our data reveal that RdRps may be more dynamic than previously thought, which would allow the execution of new functions.

## 2. Materials and Methods

### 2.1. Cells

Rabbit kidney (RK-13) cells (European Collection of Authenticated Cell Culture, Porton Down Salisbury, Wiltshire, UK) were grown in Eagle’s minimal essential medium (EMEM) (Sigma-Aldrich, St. Louis, MO, USA) supplemented with 10% foetal bovine serum (Sigma-Aldrich), 2 mM Glutamax (Gibco, Thermo Fisher Scientific, Waltham, MA, USA), 100 µg/mL of streptomycin (Gibco) and 100 units/mL of penicillin (Gibco).

### 2.2. Plasmids

The construct used for the expression of a C-terminally myc-tagged full-length RHDV RdRp was described previously [27]. This plasmid (full-length RHDV RdRp cloned into a pcDNA3.1/myc-His(-)C expression vector) was used as a PCR template to generate a set of constructs which express truncated RdRps and RdRp variants with a C-terminal myc-tag.

DNA fragments encoding N- (amino acids 1–188) and C-terminal (amino acids 211–516) parts of RHDV RdRp without the putative membrane-interacting motif (_189_LLWGCDVGVAVCAAAV-FHNICY_210_) were amplified using Platinum Taq (Life Technologies, Carlsbad, CA, USA) or Phusion DNA polymerase (Finnzymes, Thermo Fisher Scientific) and gene-specific primers (Sigma-Aldrich or GeneWorks, Thebarton, SA, Australia; for primer sequence information, see Table 1) that contained *Not*I (forward) and *Bam*HI (reverse) restriction sites. Resulting amplicons were cloned into the pcDNA3.1/myc-His(-)C expression vector (Life Technologies). To generate a construct encoding the C-terminal section (amino acids 179–516) containing the putative interacting motif (_189_LLWGCDVGVAVCAAAVFHNICY_210_), the respective DNA region was amplified using the primers with *Bam*HI restriction sites (Table 1).

To generate a construct encoding the N-terminal 268 amino acids of RHDV RdRp including the putative interacting motif, the template RdRp construct [27] was digested with *Eco*RV restriction enzyme (Promega, Madison, WI, USA) to remove the C-terminal DNA fragment encoding 248 amino acids of the protein. The product was purified and re-ligated to obtain a plasmid encoding the first 268 amino acids of RdRp in frame with a myc-tag.

The putative interacting motif along with 20 flanking amino acids (LDKVKEGKKR (N-terminus) and ELKMVARFGP (C-terminus)) was fused at its C-terminus to a myc-tag. The DNA encoding the respective fragment was amplified using primers containing *Bam*HI (forward) and *Hind*III (reverse) restriction sites (Table 1) and cloned into the pcDNA3.1/myc-His(-)C expression vector.

The putative interacting motif with 20 flanking amino acids was also fused at its C-terminus to the green fluorescent protein (GFP). The DNA encoding the respective fragment was amplified using the primers containing *Hind*III (forward) and *Bam*HI (reverse) restriction sites and cloned into pEGFP-N1 plasmid (Clontech, Mountain View, CA, USA) (Table 1).

The construct used for the expression of a C-terminally myc-tagged full-length rabbit calicivirus (RCV) RdRp was described previously [28].

The generation of plasmids containing coding sequences of MNV-1 [29], Sapovirus (SaV) (GI.1 Mc114) [30] and human norovirus (NoV) (GII.4 Sydney 2012) [31,32] RdRps was described previously. The protease–polymerase fusion protein of feline calicivirus (FCV) (strain Urbana) was synthesised and cloned into pOA-RQ expression vector (Life Technologies). The DNA fragments encoding SaV, NoV and FCV RdRps were amplified and subcloned into the pcDNA3.1/myc-His(-)C expression vector (Life Technologies) using gene-specific primers (Sigma-Aldrich) containing *Not*I (forward) and *Bam*HI (reverse) restriction sites (for primer sequence information, see Table 1).

To generate a set of RHDV RdRp variants with a less hydrophobic interaction motif, site-directed mutagenesis of double-stranded plasmid DNA was performed as described previously [28] using a full-length RHDV RdRp sequence cloned into pcDNA3.1/myc-His(-)C as a PCR template [27] and a set of specifically designed primers to introduce valine-to-serine substitutions in the newly identified interacting motif (aa 189–210) (Table 1).

The integrity of all constructs was confirmed by Sanger sequencing at the Australian Cancer Research Foundation (ACRF) Biomolecular Resource Facility, located at the Australian National University (Canberra, ACT, Australia).

### 2.3. Reference Sequences

The amino acid numbering throughout the text and figures follows the numbering of the full-length RHDV RdRp sequence (UniProtKB ID P27411).

### 2.4. Antibodies

Monoclonal mouse anti-myc (M4439), polyclonal rabbit anti-GFP (SAB4301138) and monoclonal mouse anti-beta actin (A5441) antibodies were purchased from Sigma-Aldrich. Polyclonal rabbit anti-giantin (ab24586) and goat anti-mouse IgG H&L horseradish peroxidase-conjugated (ab6789) antibodies were purchased from Abcam (Cambridge, UK). Goat anti-mouse IgG AlexaFluor 555 antibodies (A-21424) were purchased from Life Technologies and goat anti-rabbit IgG DyLight 488 antibodies (GTX76757) were purchased from GeneTex (Irvine, CA, USA). All primary and secondary antibodies were used at 1:1000 dilution.

### 2.5. Immunofluorescence

Cells grown on glass coverslips were transfected with expression constructs using Lipofectamine 3000 (Life Technologies). After a 24-h incubation period, cells were fixed with 4% formaldehyde (Polysciences, Warrington, PA, USA) in phosphate-buffered saline (PBS) for 15 min, permeabilised with 0.25% Triton X-100 in PBS for 15 min and incubated for 1 h with blocking solution containing 5% (*v*/*v*) bovine serum albumin (Sigma-Aldrich) in PBS. Primary and secondary antibodies diluted in PBS were incubated for 1 h and 30 min, respectively. Cell nuclei were stained with 4′,6-diamidino-2-phenylindole (DAPI) (Sigma-Aldrich). Coverslips were mounted onto glass slides with Fluoromount aqueous mounting medium (Sigma-Aldrich). Images were acquired on a Nikon Ti Eclipse confocal laser-scanning microscope (Nikon Instech, Tokyo, Japan) equipped with a 60× objective and analysed using ImageJ software [33].

### 2.6. Cell Counting

Subcellular localisation profiles and/or redistribution of Golgi membrane markers in cells expressing proteins of interest were studied in at least three independent experiments for each construct. When only one subcellular localisation profile of the protein was observed in all the experiments, estimated numbers are given. When several distinct staining profiles were found on the same glass cover slip, at least 20 view fields for every experiment were examined for a quantitative analysis. Cells were counted using a Nikon Eclipse Ti-U fluorescence microscope equipped with a 20× objective.

Subcellular localisation profiles of the recombinant proteins were evaluated in 1551 cells expressing full-length RHDV RdRp, 1227 cells expressing full-length RCV RdRp, 376 cells expressing SaV RdRp, and 389 cells expressing the N-terminal RDHV RdRp part with the putative interacting motif. Golgi membrane marker redistribution was evaluated in 963 cells expressing the V195S/V197S variant of RHDV RdRp, 790 cells expressing the V195S/V197S variant, and 839 cells expressing the V195S/V197S/V195S/V197S variant.

### 2.7. Hydrophobicity Scales, Prediction and Protein Structure Analysis

Kyte–Doolittle hydrophobicity plots [34], MPex [35], TM Finder [36] and TMpred [37] prediction programs were used for identification of a putative interacting motif.

Graphs were generated using GraphPad Prism software (La Jolla, CA, USA; http://www.graphpad.com/scientific-software/prism/).

Protein structures were analysed using Discovery Studio software (BIOVIA, San Diego, CA, USA; http://accelrys.com/products/collaborative-science/biovia-discovery-studio/).

### 2.8. Molecular Dynamics

Molecular dynamics simulations were carried out on the RHDV RdRp structure from PDB ID: 1KHW using Amber16 [38] employing the ff14SB forcefield. The Langevin thermostat was used for temperature regulation and long-range electrostatic interactions were treated with the particle mesh Ewald method beyond 12 Å. Constant pressure was employed using the Berendsen barostat with isotropic position scaling (NTP = 1) and a pressure relaxation time of 2 ps. The system was initially minimised to relax high-energy contacts while restraining the protein backbone, then the simulation was carried out at 298 K for 100 ns employing a 2-fs time step with SHAKE constraints applied to all bonds involving hydrogen atoms [39] and a non-bonded distance cutoff of 12 Å. The two catalytic Mn^2+^ cations were removed prior to the calculations. The trajectory was analysed using visual molecular dynamics (VMD) [40].

## 3. Results

### 3.1. Subcellular Localisation of Recombinant Calicivirus RdRps

**Figure 2:**
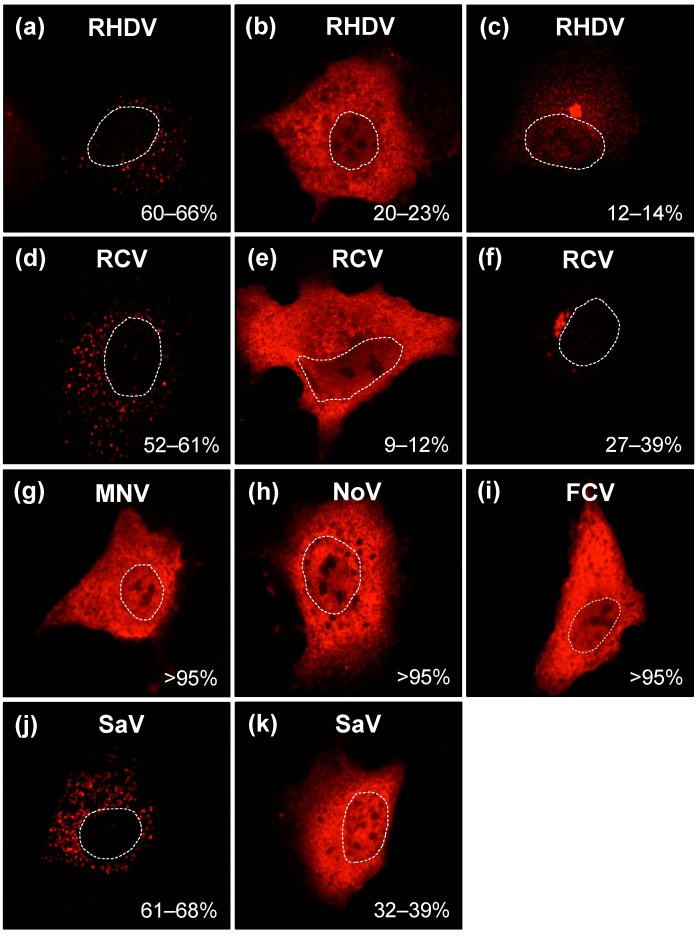
Expression of recombinant calicivirus RdRps in cultured cells. Rabbit kidney (RK-13) cells were transiently transfected with constructs encoding myc-tagged RdRp of *Rabbit haemorrhagic disease virus* (RHDV) (**a**–**c**); *Rabbit calicivirus* (RCV) (**d**–**f**); *Murine norovirus* (MNV) (**g**); *Human norovirus* (NoV) (**h**); *Feline calicivirus* (FCV) (**i**) and *Sapovirus* (SaV) (**j**,**k**). Cells were fixed 24 h after transfection and recombinant RdRps were immunostained using myc-specific antibodies. Dotted lines indicate the outline of cell nuclei. Percentages indicate frequencies with which different localisation profiles were found.

Recombinant myc-tagged RdRps of RHDV and other caliciviruses (MNV, NoV and FCV) were expressed in RK-13 cells. Cells were fixed 24 h post transfection, proteins were stained using myc-specific antibodies and subcellular localisation patterns of recombinant RdRps were evaluated. The majority of transfected cells (60–66%) accumulated RHDV RdRp as numerous distinct spots in the cytoplasm (Figure 2a). In 20–23% of transfected cells, RHDV RdRp was found in both the cytoplasm and the nucleus and was evenly dispersed within the cell (Figure 2b). In a smaller proportion of cells (12–14%), RHDV RdRp proteins accumulated in a cluster near the nucleus (Figure 2c). The RCV RdRp showed a similar subcellular localisation profile to the closely related RHDV virus: in 52–61% of transfected cells, the RCV RdRp accumulated in multiple foci in the cytoplasm (Figure 2d); in 9–12% of cells it was diffusely distributed within the nucleus and the cytoplasm (Figure 2e); in the remaining 27–39% of cells, it accumulated in a large cluster near the nucleus (Figure 2f). In contrast, cells expressing the more distantly related MNV, NoV and FCV RdRps exhibited only a single localisation pattern, i.e., RdRps were diffusely distributed within the cytoplasm and the nucleus of the cells and did not associate with any particular subcellular structures (Figure 2g–i). Finally, the SaV RdRp accumulated either as numerous spots in the cytoplasm of transfected cells (61–68%; Figure 2j) or it was evenly dispersed within the cell (32–39%; Figure 2k). The observed differences in the subcellular localisation of RdRps are not likely the result of differences in protein stability, since Western blot analysis of RHDV and MNV RdRps did not detect the accumulation of major degradation products (data not shown).

### 3.2. Identification of a Putative Motif That Is Important for the Subcellular Localisation of RHDV RdRp

Considering that the major localisation profile of RHDV RdRp resembles the staining pattern typical of intracellular vesicles, we hypothesised that RHDV RdRp may possess a motif capable of associating with intracellular membranes. Proteins can interact with membranes either indirectly via other membrane binding proteins, or directly via specific lipid-binding domains, covalently bound lipid anchors, electrostatic interactions and various non-specific hydrophobic associations [41,42,43,44]. We evaluated the presence of hydrophobic regions in the RHDV RdRp as a means of identifying putative membrane-associated motifs. Since the MNV RdRp did not associate with any particular subcellular structures, the MNV RdRp sequence was included in the analysis to determine hydrophobic motifs unique to the RHDV RdRp.

**Figure 3:**
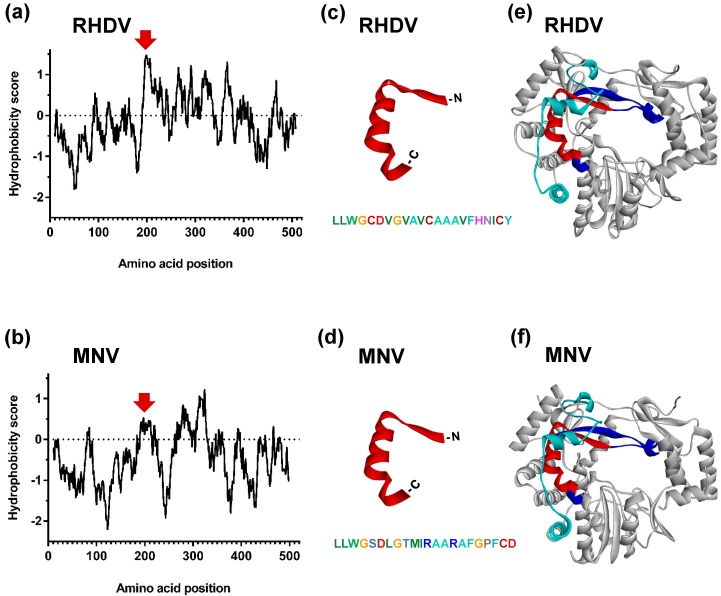
Identification of a putative membrane interacting motif in the RHDV RdRp by sequence and structure analysis. Kyte–Doolittle hydrophobicity plots for RHDV RdRp (**a**) and MNV RdRp (**b**) sequences show the presence of a relatively hydrophobic motif unique to the RHDV RdRp (positions of the hydrophobic motif in RHDV and corresponding region in MNV are indicated by red arrows). Amino acid sequence and secondary structure of the putative membrane interacting motif of the RHDV RdRp (**c**) and the corresponding region in MNV (**d**). Location of proposed membrane interacting motif within the tertiary structure of the RHDV RdRp (**e**) and location of the corresponding region in MNV RdRp (**f**). The putative functional motif is shown in red, and the location of the F and G homomorphs is shown in dark blue and cyan, respectively. Protein Bank Database IDs of crystal structures of RdRps: 1KHW (RHDV) and 3UQS (MNV).

Kyte–Doolittle hydrophobicity plots showed that the RHDV RdRp does not have any highly hydrophobic regions near the C- and N-terminus that may serve as transmembrane anchors (Figure 3a). However, both MNV and RHDV RdRps contain a relatively hydrophobic region located between amino acid positions 250–400, a protein section that is involved in the formation of the palm subdomain (Figure 3a,b). In addition to this hydrophobic region common to both RdRps, the RHDV RdRp has an additional relatively hydrophobic stretch of amino acid residues (_189_LLWGCDVGVAVCAAAVFHNICY_210_) (Figure 3a,c) that is located next to the conserved F motif and forms the C-terminal part of the F homomorph (Figure 3e). This putative membrane-interacting motif is partially buried, suggesting that it is highly likely that any putative interaction with intracellular membranes involves only one side of a membrane bilayer, if the RdRp can indeed associate directly with membranes. While the high hydrophobicity is a primary characteristic of membrane-interacting motifs, other factors such as helicity, amino acid composition and solvent accessibility are also important [35,36,45,46]. Although this newly identified hydrophobic motif is largely hidden underneath the G homomorph, C-terminal residues that form an α-helix are partially exposed to the exterior surface.

We also analysed the hydrophobicity profiles of RdRps from caliciviruses other than RHDV and compared their subcellular localisation patterns in RK-13 cells. We found that the RCV RdRp also possesses a relatively hydrophobic stretch of amino acids in the F homomorph, exactly in the same position as the putative interacting motif in the RHDV RdRp (data not shown). Interestingly, the RCV RdRp showed a similar intracellular localisation profile as RHDV, which included the ability to accumulate as particular cytoplasmic spots (Figure 2d–f). The RdRps of NoV and MNV had comparable hydrophobicity and subcellular localisation profiles but were not able to associate with any particular structures in the cell (Figure 2g,h). In the case of the FCV RdRp, Kyte–Doolittle hydrophobicity plotting indicated the presence of a relatively hydrophobic motif in F homomorph; but prediction programs that consider parameters in addition to hydrophobicity did not point to the respective sequence as a candidate for membrane association (data not shown). When FCV RdRp was expressed in RK-13 cells, the protein was diffusely distributed through the cytoplasm and the nucleus, similar to MNV and NoV RdRps (Figure 2g–i). For the SaV RdRp, some of the prediction programs, e.g., TM-finder, suggested a putative transmembrane segment in C-terminal part of the F homomorph at the same position as the putative interacting motif in the RHDV RdRp (data not shown). Most interestingly, when expressed in RK-13 cells, the SaV RdRp accumulated in multiple cytoplasmic locations in up to 70% of transfected cells (Figure 2j,k), a pattern reminiscent to that of RHDV and RCV RdRps (Figure 2a,d). Taken together, our observations suggest a correlation between the presence of a hydrophobic motif in the F homomorph of calicivirus RdRps and a particular subcellular localisation profile.

### 3.3. Role of the Proposed Hydrophobic Motif in Regulating the Subcellular Localisation of RHDV RdRp

**Figure 4:**
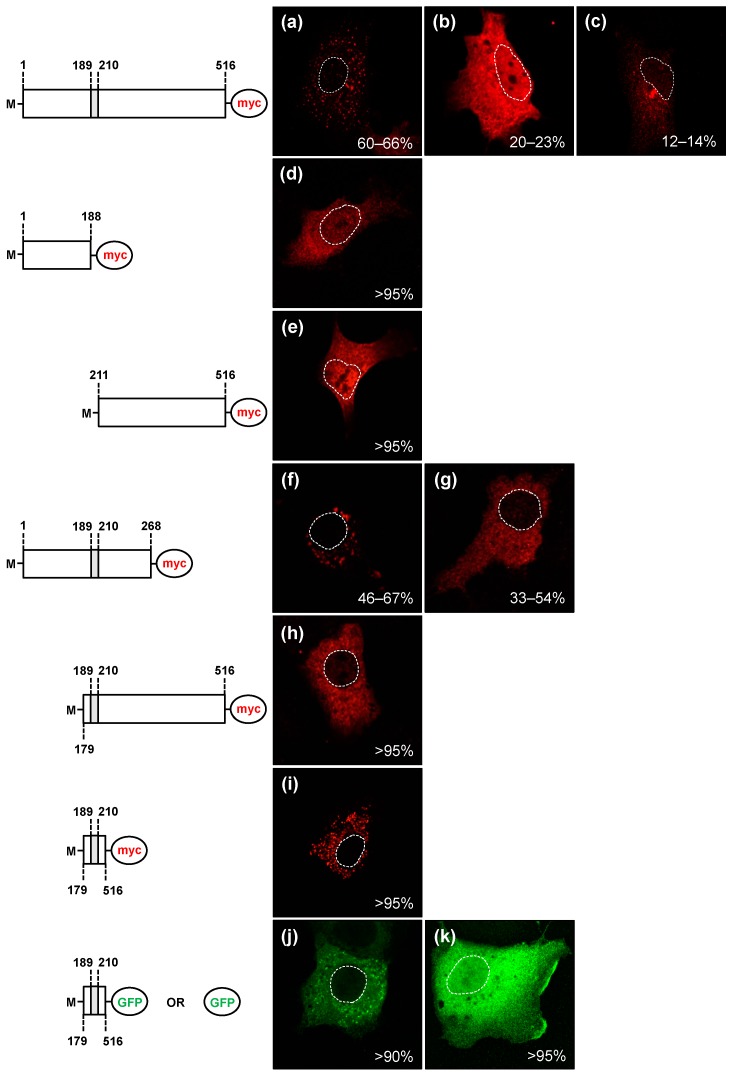
The newly identified hydrophobic motif is important for the subcellular localisation of RHDV RdRp and sufficient to change the subcellular localisation of GFP. RK-13 cells were transiently transfected with expression constructs coding for myc-tagged versions of the full-length RHDV RdRp (**a**–**c**); the N-terminal part of the protein without the hydrophobic motif (**d**); the C-terminal part without the motif (**e**); the N-terminal part with the motif (**f**,**g**); the C-terminal part with the motif (**h**); or the motif (and a few flanking amino acids) without the remainder of the protein (**i**). Furthermore, cells were transfected with constructs in which the motif was fused to GFP (**j**); or control constructs for the expression of unaltered GFP (**k**). Cells were fixed 24 h after transfections and recombinant proteins were immunostained using anti-myc or anti-GFP antibodies. Schematic representations of the various recombinant proteins are shown to the left of the fluorescence images. A grey box indicates the position of the hydrophobic motif. M indicates an additional N-terminal methionine. Dotted lines indicate the outline of cell nuclei. Percentages indicate frequencies with which different localisation profiles were found.

To explore the role of the proposed motif in regulating the subcellular localisation of the RHDV RdRp, we expressed a set of truncated proteins with and without the putative interacting motif _189_LLWGCDVGVAVCAAAVFHNICY_210_. Recombinant myc-tagged versions of the truncated proteins were expressed in RK-13 cells, fixed 24 h post transfection and stained using myc-specific antibodies. In cells expressing N- or C-terminal parts of RHDV RdRp without the putative interacting motif, the recombinant proteins were diffusely distributed in the nucleus and cytoplasm (Figure 4d,e). These truncated proteins clearly lacked the ability to accumulate in distinct cytoplasmic foci (compare Figure 4a–c and Figure 4d,e). In contrast, when the N-terminal two-thirds of the protein (containing the putative interacting motif) were expressed, 46–67% of transfected cells still accumulated the recombinant protein as distinct spots in the cytoplasm (Figure 4f); in the remaining 33–54% of cells, the truncated protein showed a more diffuse distribution in the cytoplasm than the full-length protein, but did not accumulate in the nucleus (Figure 4g). Similarly, a C-terminal peptide with the putative interacting motif accumulated exclusively in the cytoplasm of transfected cells (Figure 4h). Although none of the truncated proteins containing the putative interacting motif could fully reproduce the subcellular localisation of the full-length RdRp, the presence of putative interacting motif changed the localisation of truncated proteins and ensured their accumulation in the cytoplasm of the cells.

When the putative interacting motif was expressed on its own (along with 10 flanking amino acids on both N- and C-termini, and 10 amino acids that belong to a small protein (myc) tag), the majority of transfected cells (up to 100%) accumulated the recombinant protein in distinct cytoplasmic foci (Figure 4i). This subcellular localisation pattern was very similar to the most prevalent localisation (as found in 60–66% of transfected cell) of the full-length protein (compare Figure 4a,i). Furthermore, when the putative interacting motif (along with 20 flanking amino acids) was fused to the GFP, the resulting fusion protein displayed a different subcellular localisation compared with the localisation of GFP alone (Figure 4j,k). Control cells expressing GFP alone showed a diffuse distribution of the proteins in the nucleus and the cytoplasm. In contrast, the majority of transfected cells (90%) that expressed the putative interacting motif:GFP fusion protein accumulated the fusion protein exclusively in the cytoplasm, suggesting that it could no longer cross nuclear pores or was actively retained in the cytoplasm.

### 3.4. Amino Acid Substitutions Decreasing the Hydrophobicity of the Putative Interacting Motif Change the Subcellular Localisation of RHDV RdRp and Reduce Its Ability to Induce Redistribution of the Golgi Membranes

Since hydrophobicity is a hallmark characteristic for membrane interacting motifs [45], we introduced a series of valine-to-serine amino acid substitutions in the putative interacting peptide of the RHDV RdRp. These substitutions were designed to reduce the hydrophobicity of the motif. Variant proteins were expressed in RK-13 cells and their subcellular localisation profiles were studied. As the expression of recombinant full-length RHDV RdRp was also able to induce a redistribution of Golgi membrane markers in the transfected cells [27], we also tested the ability of variant RdRps to induce a redistribution of Golgi membrane markers in transfected cells by simultaneously staining recombinant proteins and Golgi membranes using anti-myc and anti-giantin antibodies, respectively.

None of the truncated RHDV RdRp proteins or fusion proteins containing the putative interacting motif with flanking amino acids and a protein tag (myc or GFP) could induce a redistribution of the Golgi membrane markers in transfected cells (data not shown). This suggests that although the presence of the newly identified motif is a pre-requisite for the observed redistribution of Golgi membranes, its presence is not sufficient to induce changes to the Golgi architecture.

**Figure 5:**
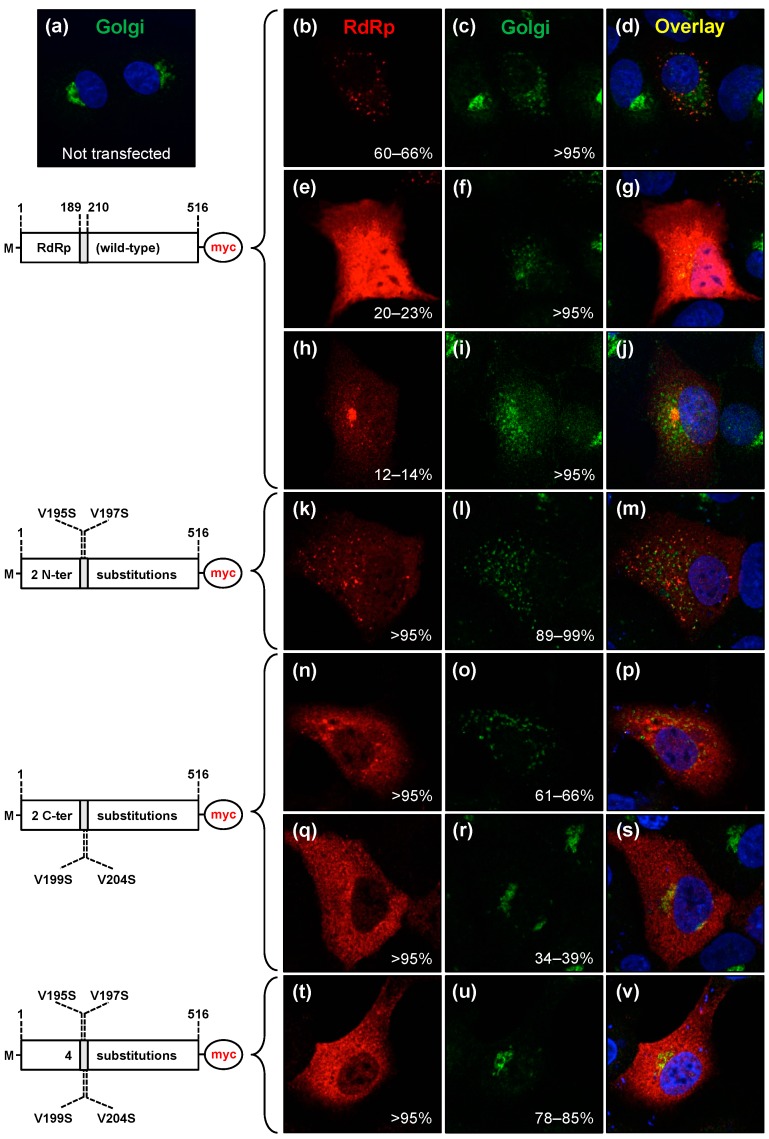
Amino acid substitutions decreasing the hydrophobicity of the newly identified motif change the subcellular localisation and the ability of the RHDV RdRp to induce Golgi membrane rearrangements. RK-13 cells were transfected with constructs encoding myc-tagged wild-type RHDV RdRp (**b**–**j**) or constructs encoding versions of the protein in which valine (V) residues within the new hydrophobic motif had been changed to serine (S) residues: i.e., V195S and V197S (**k**–**m**); V199S and V204S (**n**–**s**); and V195S, V197S, V199S, and V204S (**t**–**v**). Cells were fixed 24 h after transfection, recombinant proteins and the Golgi membrane marker giantin were immunostained using myc-specific antibodies (shown in red) and anti-giantin antibodies (shown in green), respectively. Cell nuclei were stained using 4′,6-diamidino-2-phenylindole (DAPI, blue). Schematic representations of the various recombinant proteins are shown to the left of the fluorescence images. A grey box indicates the position of the hydrophobic motif. M indicates an additional N-terminal methionine. Compared with the giantin staining in untransfected control cells (**a**), the majority of cells expressing wild-type RdRp or proteins with the two N-terminal (relative to the hydrophobic domain) substitutions V195S and V197S showed a giantin staining indicative of rearranged Golgi membranes (**c**,**f**,**i**,**l**). RdRp variants with the C-terminal substitutions V199S and V204S were no longer able to change Golgi membrane in a about a third of cells (**o**,**r**) and variants with all four substitutions did not change Golgi membranes in the majority of cells (**u**).

In cells expressing wild-type RHDV RdRp, three different subcellular localisation profiles were found and Golgi membrane redistribution was observed in almost all cells irrespective of the RdRp subcellular localisation pattern (Figure 5b–j).

In contrast to the wild-type protein, a variant RdRp containing two valine-to-serine substitutions at position 195 and 197 in the N-terminal and less exposed part of the putative interacting motif (V195S/V197S) exhibited only one subcellular localisation profile. The variant protein accumulated in the cytoplasm where most of the signal came from widely distributed distinctive spots (Figure 5k). In the majority of cells expressing this protein, the Golgi membrane marker giantin was redistributed, indicating that the V195S/V197S variant was still able to induce changes to Golgi membranes (Figure 5k–m).

A variant RdRp containing two valine-to-serine substitutions in the C-terminal and more exposed part of putative interacting motif (V199S/V204S) also exhibited only one subcellular localisation profile (Figure 5n,q). However, this protein largely lost the ability to accumulate as particular subcellular spots that are characteristic for wild-type RdRp and the V195S/V197S variant. This phenotype correlated with a diminishing ability to induce Golgi membrane rearrangements. Only two-thirds of transfected cells (61–66%) showed dispersed Golgi membranes (Figure 5n–p), while the remaining cells (34–39%) exhibited a Golgi staining similar to that of untransfected control cells (compare Figure 5q–s and Figure 5a).

The subcellular localisation profile of a variant RdRp containing four valine-to-serine substitutions (V195/S197S/V199S/V204S) did not differ significantly from the V199S/V204S variant, i.e., V195/S197S/V199S/V204S variant did not accumulate as specific cytoplasmic spots (Figure 5t). However, in contrast to the V195S/V197S and V199S/V204S variants, V195S/V197S/V199S/V204S variant almost completely lost its ability to induce changes in the localisation of a Golgi membrane marker (78–85% cells showed a normal Golgi staining; Figure 5t–v).

Taken together, our results indicate that the putative interacting motif _189_LLWGCDVGVAVCAAAVFHNICY_210_ is important for the subcellular localisation of the RdRp and its ability to induce a redistribution of Golgi membranes.

### 3.5. Molecular Dynamics

**Figure 6:**
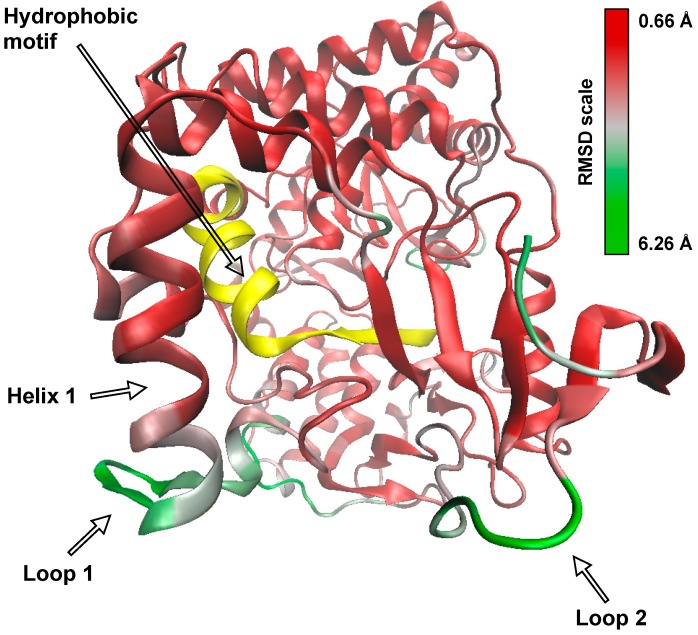
Backbone root-mean-square deviation (RMSD) per residue over time for the 100-ns RHDV RdRp molecular dynamics trajectory. Red and green indicate stability and mobility, respectively. The hydrophobic motif is shown in yellow for structural perspective, but was stable throughout the simulation.

**Figure 7:**
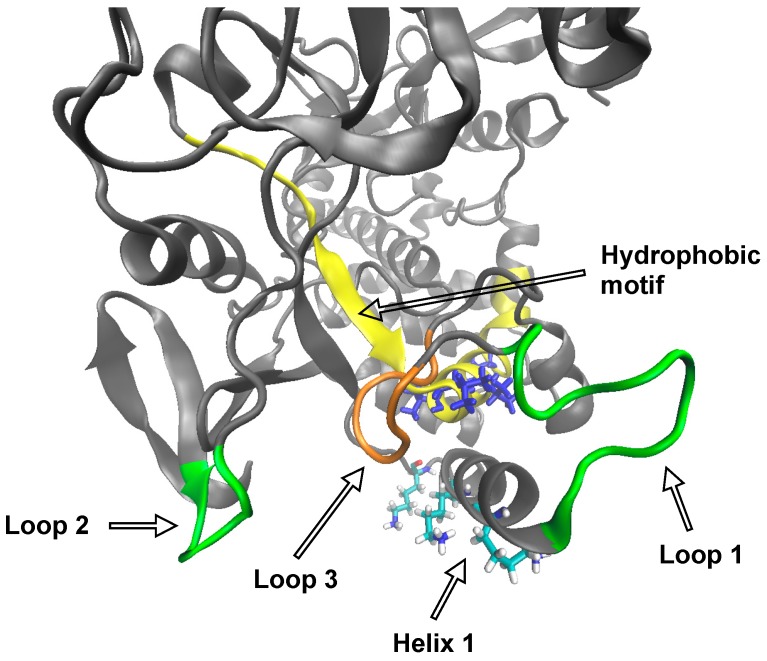
Structural features proposed to be involved in interactions with the Golgi membranes, with Loop 1 and Loop 2 shown in green, Loop 3 shown in orange and the hydrophobic motif shown in yellow. Val195, Val197, Val199 and Val204 on the hydrophobic motif are shown in blue stick representation. The three collinear lysines (Lys 149, Lys 153 and Lys 162) on Helix 1 are shown as cyan atom-in-stick representations.

We carried out a 100-ns molecular dynamics simulation of RHDV RdRp with the goal of revealing potential mechanisms by which the hydrophobic motif might become exposed to the inner membrane. An examination of the root-mean-square deviation (RMSD) per residue showed four main regions of significantly higher mobility (excluding the termini, which are typically mobile in almost all proteins) throughout the simulation compared with the rest of the protein (Figure 6). Interestingly, two of these mobile regions are loops that flank the opening through which the hydrophobic motif could be accessed. The first of these loops (labelled Loop 1 in Figure 6 and Figure 7; consisting of residues 135–145) starts at the base of a helix (Helix 1) and contains three collinear, positively charged lysine residues (Lys 149, Lys 153 and Lys 162). The second loop (Loop 2; residues 20–25) is hydrophobic, solvent-exposed. These two labile loops flank a partially solvent-exposed hydrophobic loop (Loop 3; residues 126–132) that ‘protects’ the hydrophobic membrane interaction motif. Based on the structural data, we propose the following plausible hypothesis for the mechanism by which the buried hydrophobic motif interacts with membranes: The positively charged edge of Helix 1 interacts with a negatively charged membrane surface, bringing Loop 1 into contact with the membrane where hydrophobic interactions draw the enzyme further towards the membrane to a point at which Loop 3 makes contact with the membrane, then allowing the hydrophobic motif to insert itself into the membrane.

## 4. Discussion

Previously, we showed that RdRps from RHDV and RCV have an unusual subcellular localisation and are able to induce a redistribution of Golgi membranes in transfected cells [27,28]. For the RHDV RdRp, a predominantly cytoplasmic localisation and the ability to influence the localisation of Golgi membranes were observed in kidney and liver cell lines of three different species [27]. An accumulation in distinct cytoplasmic spots was observed in all three cell lines, but subtle differences existed (e.g., Vero cells showed a more defuse staining compared with RK-13 and Huh-7 cells), suggesting that tissue and/or species-specific factors play a role in the intracellular trafficking and accumulation of the RHDV RdRp. However, no such characteristics (i.e., the accumulation of distinct cytoplasmic spots and the impact on Golgi membranes) were reported for RdRps of more distantly related caliciviruses, such as MNV [22]. Our own findings are in line with previous reports; the expression of NoV, MNV, and FCV RdRps did not affect the localisation of Golgi membrane markers (data not shown). Furthermore, although we observed that the SaV RdRp showed intracellular accumulation patterns similar to those of lagovirus RdRps (Figure 2j), its expression did not induce detectable changes in the localisation of Golgi membranes (data not shown). It remains to be seen whether RdRps from SaV and other caliciviruses possess functional interaction domains and influence the localisation of cellular membranes in a more subtle manner than lagovirus RdRps.

Since the RHDV RdRp does not have additional domains if compared with related enzymes, e.g., MNV RdRp, the structural basis of the unusual ability of the RHDV RdRp to induce a redistribution of Golgi-membranes is intriguing. We hypothesised that the RHDV RdRp may interact with subcellular membranes directly via hydrophobic motif(s) but Kyte–Doolittle hydrophobicity plots [34] showed that there were no highly hydrophobic sequences near the N- and C-terminus that could serve as transmembrane anchors. However, we detected the presence of a relatively hydrophobic helix in the middle of the protein, suggesting that RHDV RdRp may act as a peripheral membrane protein. An interaction with only one membrane leaflet would not require transmembrane spanning segments. Nevertheless, we analysed the RHDV RdRp sequence for putative transmembrane domains, using programs such as MPex [35], TM Finder [36] and TMpred [37]. These prediction programs consider parameters in addition to hydrophobicity in their analytical algorithms to improve prediction results. Since membrane-interacting motifs of peripheral membrane proteins share a number of properties with bona fide transmembrane segments [45,46], the prediction programs pointed (with a low probability) to the proposed interacting motif as a putative transmembrane segment (data not shown).

In accordance with our prediction that RHDV RdRp associates with membranes directly via a hydrophobic motif in the F homomorph, truncated proteins accumulated in different subcellular compartment depending on the presence of absence of the hydrophobic motif. Truncated proteins still containing the proposed interacting motif changed the subcellular localisation and accumulated exclusively in the cytoplasm of the cells. However, it should be noted that none of the truncated proteins containing the proposed interacting motif could fully reproduce the localisation pattern of the full-length RdRp (compare Figure 4a–c with Figure 4d–i). Similarly, when fused to GFP, the proposed motif was sufficient to change the localisation of the resulting fusion protein, although the staining pattern differed from that of the full-length RdRp. These observations are similar to the results obtained for the Semliki Forest virus (SFV) nsP1 protein, which associates with membranes in a monotopic manner through a hydrophobic peptide and a subsequent palmitoylation to tighten membrane association [47]. These experiments with SFV nsP1 showed that its membrane binding peptide, which is located in the middle of the protein, is essential for membrane association; however, when fused to GFP, at least two tandem copies of the peptide were required to change the localisation of GFP and even then, the fusion protein could not fully reproduce the staining pattern of full-length nsP1 [47]. It is tempting to speculate that, similar to the membrane binding peptide of the SFV nsP1 protein, the hydrophobic motif of RHDV RdRp provides affinity for membranes, but other parts of the RdRp may assist in attaching the protein more firmly to cellular membranes or to membrane-associated proteins. Such a scenario is in line with the notion that membrane targeting of peripheral membrane proteins usually relies on more than a single mechanism [44].

Previously, we showed that mutations in the active site of the RHDV RdRp (GDD→GND and GDD→GAA) that destroy enzymatic activity do not change the subcellular localisation and the ability of the RdRp to induce Golgi membrane rearrangements [28]. In contrast, valine-to-serine amino acid substitutions that reduced the hydrophobicity of the putative interacting motif did not only affect the subcellular localisation of the RHDV RdRp but also decreased its ability to induce a redistribution of Golgi membrane markers. These observations support the idea that the proposed motif may interact directly with lipid bilayers, since hydrophobicity is a primary characteristic of membrane-targeting α-helixes [45].

Interestingly, the putative interacting motif was located within the C-terminal part of the conserved F homomorph of the core polymerase domain of RHDV RdRp. This observation complements previous reports, which suggest that the core polymerase domain serves additional function(s) beyond its well-established activities in replicating RNA. For example, RdRps of some picornaviruses, such as *Foot-and-mouth disease virus* (FMDV), *Poliovirus* (PV) and *Enterovirus* 71 (EV71) enter the nucleus due to the presence of nuclear localisation signals (NLS) and alter cellular gene expression [26,48,49,50]. In case of FMDV, the NLS is located N-terminally in the fingers subdomain [48,49], while in PV and EV71 RdRps, NLSs are found within the G homomorph, downstream from the C-terminal edge of the G motif [26,50].

The molecular dynamics simulation provided compelling evidence that the structural motifs flanking the surface leading into the hydrophobic motif are highly labile and could give way upon association with the Golgi membrane surface. This would provide access to the hydrophobic core of the membrane, first to the already partially exposed hydrophobic Loop 3, and then to the hydrophobic motif itself. This model provides a series of potential targets for future mutagenesis studies. For example, it would be interesting to study how reducing the hydrophobicity of Loop 3 or altering the positive charges on Helix 1 affect the interaction of the polymerase with Golgi membranes. Determining the precise mechanism of membrane binding could lead to the development of new drugs for related viral polymerases of interest in human health that, based on their sequences/structures, are likely to have similar membrane associations.

## 5. Conclusions

The RHDV RdRp is associated with distinct, but yet uncharacterised subcellular structures and is capable of inducing a redistribution of Golgi membranes. Here, we describe a functional motif in RHDV RdRp that is important for the subcellular localisation of the protein. Amino acid substitutions decreasing the hydrophobicity of the newly described motif not only changed the subcellular localisation of the protein, but also reduced its ability to induce rearrangements of the Golgi membrane markers. Further extension of this data to determine the role of this motif in RHDV replication is highly difficult because of the lack of a robust cell culture system for rabbit calicivirus propagation. Our findings supplement previous reports suggesting that the core polymerase domain of some viral RdRps may serve additional functions beyond the well-established role in replicating RNA.

## Figures and Tables

**Table 1:** Primers used to generate variant RHDV RdRp proteins.

Vector	Construct	Primer Sequence (5′→3′)
pcDNA 3.1/myc-His(-)C	pRHDV-RdRp-Nter	F: ATCGTTATAGCGGCCGCCTGCAGTAGCCACCATGACGTCAAACTTCTTCTGTGG R: TATGGGATCCTCTTCTTTCCCTCCTTCACCTTGTCAAG
	pRHDV-RdRp-Cter	F: TATGGCGGCCGCGCCACCATGTACGAACTAAAGATGGTCGCGCGG R: ATCGTCATCGGATCCAGATATCCTCCATAACATTCACAAATTCGTC
	pRHDV-RdRp-Cter-PIM	F: TATGGGATCCTAGCCACCATGCTTGACAAGGTGAAGGAGGGAAAGAAG R: ATCGTCATCGGATCCAGATATCCTCCATAACATTCACAAATTCGTC
	pRHDV-FAA-PIM-FAA:myc	F: TATGGGATCCTAGCCACCATGCTTGACAAGGTGAAGGAGGGAAAGAAG R: TATCAAGCTTCGGGGCCAAACCGCGCGACCATC
	pMNV-RdRp	F: TATGGCGGCCGCCACCATGCTTCCCCGCCCCTC R: TATGGGATCCTCTCATCCTCATTCACAAAG
	pSaV-RdRp	F: TATGGCGGCCGCCACCATGGATGAATTCCAATGGAAGGG R: TATGGGATCCTCTCCATCTCAAACACTATTTTG
	pNoV-RdRp	F: TATGGCGGCCGCCACCATGGGTGGTGACAGTAAGGG R: TATGGGATCCTTTCGACGCCATCTTCATTC
	pFCV-RdRp	F: TATGGCGGCCGCCACCATGGTGACAGCCCAGAAATATGATG R: TATGGGATCCTCACTTCAAACACATCACAATGC
pEGFP-N1	pRHDV-FAA-PIM-FAA:GFP	F: TATGAAGCTTTAGCCACCATGCTTGACAAGGTGAAGGAGGGAAAGAAG R: TATCGGATCCTCGGGGCCAAACCGCGCGACCATC
pcDNA 3.1/myc-His(-)C	pRHDV-V195S-V197S	F: CCGTGTGTGCTGCCGCAGTTTTC R: CG**GA**ACCA**GA**GTCACAGCC
	pRHDV-V199S-V204S	F: CC**TC**GTGTGCTGCCGCA**TC**TTTC R: CGACACCAACGTCACAGCC
	pRHDV-V195S-V197S-V199S-V204S	F: CC**TC**GTGTGCTGCCGCA**TC**TTTC R: CG**GA**ACCA**GA**GTCACAGCC

F—forward primer; R—reverse primer; Cter—C-terminus; Nter—N-terminus; FAA—flanking amino acids; PIM—putative interacting motif; GFP—green fluorescent protein; EGFP—enhanced green fluorescent protein. Restriction sites are underlined. Nucleotide substitutions are in bold.
